# Risk Model for Colorectal Cancer in Spanish Population Using Environmental and Genetic Factors: Results from the MCC-Spain study

**DOI:** 10.1038/srep43263

**Published:** 2017-02-24

**Authors:** Gemma Ibáñez-Sanz, Anna Díez-Villanueva, M. Henar Alonso, Francisco Rodríguez-Moranta, Beatriz Pérez-Gómez, Mariona Bustamante, Vicente Martin, Javier Llorca, Pilar Amiano, Eva Ardanaz, Adonina Tardón, Jose J. Jiménez-Moleón, Rosana Peiró, Juan Alguacil, Carmen Navarro, Elisabet Guinó, Gemma Binefa, Pablo Fernández Navarro, Anna Espinosa, Verónica Dávila-Batista, Antonio José Molina, Camilo Palazuelos, Gemma Castaño-Vinyals, Nuria Aragonés, Manolis Kogevinas, Marina Pollán, Victor Moreno

**Affiliations:** 1 Cancer Prevention and Control Program, Catalan Institute of Oncology-IDIBELL, L’Hospitalet de Llobregat, Spain; 2 CIBER Epidemiología y Salud Pública (CIBERESP), Madrid, Spain; 3 Gastroenterology Department, Bellvitge University Hospital-IDIBELL, L’Hospitalet de Llobregat, Spain; 4 Environmental and Cancer Epidemiology Department, National Center of Epidemiology - Instituto de Salud Carlos III, Madrid, Spain; 5 Oncology and Hematology Area, IIS Puerta De Hierro, Cancer Epidemiology Research Group, Madrid, Spain; 6 ISGlobal Centre for Research in Environmental Epidemiology (CREAL), Barcelona, Spain; 7 Instituto de Biomedicina (IBIOMED). Grupo de investigación en interacciones gen ambiente y salud. Universidad de León, León, Spain; 8 Universidad de Cantabria - IDIVAL, Santander, Spain; 9 Public Health Division of Gipuzkoa, Biodonostia Research Institute, San Sebastian, Spain; 10 Navarra Public Health Institute, Navarra, Spain; 11 University Institute of Oncology of Asturias (IUOPA), Universidad de Oviedo, Oviedo, Spain; 12 Instituto de Investigación Biosanitaria de Granada (ibs.GRANADA), Hospitales Universitarios de Granada, Granada, Spain; 13 Fundación para el Fomento de la Investigación Sanitaria y Biomédica de la Comunitat Valenciana FISABIO–Salud Pública, Valencia; 14 Centre for Research in Health and Environment (CYSMA), Universidad de Huelva, Huelva, Spain; 15 Department of Epidemiology, Murcia Regional Health Council, IMIB-Arrixaca and Department of Health and Social Sciences, Universidad de Murcia, Murcia, Spain; 16 IMIM (Hospital Del Mar Medical Research Institute), Barcelona, Spain; 17 Universitat Pompeu Fabra (UPF), Barcelona, Spain; 18 School of Public Health, Athens, Greece; 19 Department of Clinical Sciences, Faculty of Medicine, University of Barcelona, Barcelona, Spain

## Abstract

Colorectal cancer (CRC) screening of the average risk population is only indicated according to age. We aim to elaborate a model to stratify the risk of CRC by incorporating environmental data and single nucleotide polymorphisms (SNP). The MCC-Spain case-control study included 1336 CRC cases and 2744 controls. Subjects were interviewed on lifestyle factors, family and medical history. Twenty-one CRC susceptibility SNPs were genotyped. The environmental risk model, which included alcohol consumption, obesity, physical activity, red meat and vegetable consumption, and nonsteroidal anti-inflammatory drug use, contributed to CRC with an average per factor OR of 1.36 (95% CI 1.27 to 1.45). Family history of CRC contributed an OR of 2.25 (95% CI 1.87 to 2.72), and each additional SNP contributed an OR of 1.07 (95% CI 1.04 to 1.10). The risk of subjects with more than 25 risk alleles (5^th^ quintile) was 82% higher (OR 1.82, 95% CI 1.11 to 2.98) than subjects with less than 19 alleles (1^st^ quintile). This risk model, with an AUROC curve of 0.63 (95% CI 0.60 to 0.66), could be useful to stratify individuals. Environmental factors had more weight than the genetic score, which should be considered to encourage patients to achieve a healthier lifestyle.

Colorectal cancer (CRC) screening by faecal occult blood testing has been demonstrated to reduce CRC incidence and mortality^1^, as well as being a cost-effective strategy compared to no screening^2^,^3^. Recent evidence of the benefit-harms balance of cancer screening has led to proposals for more personalized strategies based on individual cancer risk. Effectiveness of a screening strategy depends on the average cancer risk of the target population. Today, the target population is defined basically by age (≥50 years old), which has been called a ‘one-size-fits-all’ strategy^4^. This strategy implies performing unnecessary screening tests in low-risk people leading to avoidable risks for patients and extra costs for the healthcare system. On the other hand, high-risk patients may receive non-invasive testing, which is a suboptimal screening technique in their case. A risk-based CRC screening that included environmental risk factors, family history of CRC, and information derived from genetic susceptibility loci could improve not only the efficacy of the screening program but also the adherence of high-risk patients when properly informed of their personal risk.

Several risk prediction models, either for CRC or advanced neoplasia, have been previously developed, all with limited discriminating ability^5^,^6^,^7^,^8^,^9^,^10^. These studies have encompassed the traditional environmental risk factors for CRC including age, sex, family history of CRC, smoking, alcohol, Body Mass Index (BMI), physical activity, diet, and some drugs (nonsteroidal anti-inflammatory drugs (NSAID), acetylsalicylic acid (ASA), calcium and vitamins). Furthermore, with the identification of CRC-associated common single-nucleotide polymorphisms (SNPs), a few studies have added genetic susceptibility information together with some of the clinical risk factors^6^,^11^,^12^,^13^,^14^. Each common low-penetrance allele is associated with a small increase in risk of CRC, but the combined effect of multiple SNPs may achieve a higher degree of risk discrimination, which could be useful to stratify the population^15^,^16^,^17^,^18^. In this study we have developed a risk stratification model that combines environmental factors with family history and genetic susceptibility. Furthermore, we have interpreted the relative contribution of these factors and the utility of the model for risk stratification and public health intervention.

## Materials and Methods

### Study population

A detailed description of the MCC-Spain case-control study has been provided elsewhere^19^. Briefly, between 2008–2013, 10183 subjects aged 20–85 years were enrolled in 23 hospitals and primary care centres in 12 Spanish provinces (Madrid, Barcelona, Navarra, Girona, Gipuzkoa, León, Asturias, Murcia, Huelva, Cantabria, Valencia, and Granada). Eligible subjects included histological confirmed incident cases of CRC (n = 2171). Potential controls that reported having had a diagnosis of CRC were excluded. Both cases and controls were free of personal CRC history. Controls were frequency-matched to cases, by age, sex, and region, ensuring that in each region there was at least one control of the same sex and a 5-year interval for each case. For the present study, a subset including 1336 CRC cases and 2744 controls with genotype data were analysed.

### Data collection

A structured computerized epidemiological questionnaire was administered by trained personnel in a face-to-face interview. Also, subjects filled in a semi-quantitative Food Frequency Questionnaire (FFQ), and blood samples and anthropometric data were obtained following the study protocol.

Only variables clearly related with CRC were considered for the development of risk models. The variables considered were: family history of CRC (none versus first or second or third-degree); cigarette smoking, grouped into non-smokers and smokers (including former and current); average alcohol consumption between 30 and 40 years of age (in standard units of alcohol, SUA), categorized into low-risk and high-risk consumption (>4 SUA/day in men and >2 SUA/day in women)^20^; BMI (calculated with the weight reported at 45 years of age), which was categorized according to World Health Organization criteria as underweight, normal weight, and overweight (<30 kg/m^2^) versus obese (≥30 kg/m^2^); average physical exercise, measured from self-reported leisure-time activity performed in the past 10 years and used to estimate the Metabolic Equivalent of Task (MET) per hour per week, calculated using the Ainsworth’s compendium of physical activities^21^, and categorized as no physical activity in leisure time (0 MET) and any physical activity in spare time (>0 MET); red meat consumption, including meat from mammals (cattle, oxen, veal, beef, pork, etc.), meat from hunting birds (duck, pheasant, etc.), organ meats (liver, brains, etc.), cured meat (ham, bacon, etc.), and processed meat (hot dogs, sausages, meat balls, etc.). High intake of red meat was considered eating ≥65 g/day; vegetables, classified as low or high intake using 200 g/day as cut-off.

All the patients’ drugs were recorded but only nonsteroidal anti-inflammatory drugs (NSAIDs) (cyclooxygenase 1 and 2 inhibitors) and ASA were taken into account for this study. Patients were considered users of NSAIDs/ASA if they consumed ≥1 times/day for at least 1 year.

The location of the CRC was defined according to its anatomic distribution: proximal colon (colon above the level of the splenic flexure, or including it), distal (descending colon and sigmoid colon), and rectum.

All procedures performed in studies involving human participants were in accordance with the ethical standards of the institutional and/or national research committee, and with the 1964 Helsinki Declaration and its later amendments or comparable ethical standards. The protocol of MCC-Spain was approved by each of the ethics committees of the participating institutions. The specific study reported here was approved by the Bellvitge Hospital Ethics Committee with reference PR 149/08. Informed consent was obtained from all individual participants included in the study.

### Genotyping

The Infinium Human Exome BeadChip (Illumina, San Diego, USA) was used to genotype >200000 coding markers plus 5000 additional custom SNPs selected from previous GWAS studies or genes of interest. The genotyping array included 25 SNPs previously identified as susceptibility variants for CRC in genome-wide association studies (GWAS)^22^. Ten SNPs were in the commercial array; we included in the custom content 15 more that had been identified at the time of designing the array (July 2012). For regions where multiple SNPs had been reported, we included only the most statistically significant SNP for each locus when linkage disequilibrium was >0.5. As a result, we included a total of 21 SNPs in the final analysis, detailed in Table 1.

### Statistical analysis

Multivariate logistic regression models were used to build risk models. All models were adjusted by a propensity score^23^ to reduce bias related to differences in case and control selection frequencies, and account for the frequency matched design of the study. The propensity score model was constructed as the individual prediction (in logit scale) of a logistic regression in which case/control status was modelled with age, sex, level of education, recruiting centre, and the first 3 principal components of genetic ancestry obtained from genotyping data. The interactions between age and sex and centre and sex were also included in the model. The propensity score was added as a continuous variable to adjust the risk models. Since age and sex were used as stratification factors for frequency matching the selection of controls, these variables cannot contribute to the risk model.

An environmental risk score (ERS) was built including all the significant covariates that can be modified (alcohol use, BMI, physical exercise, red meat and vegetable intake, and NSAIDs/ASA use). Family history was not considered in this environmental score since it is not modifiable, and its effect was assessed as a separate factor. Missing values in variables were imputed using the expected value derived from a model built with complete cases. For categorical variables, the most frequent value was imputed.

To assess genetic susceptibility, an additive genetic risk score (GRS) was put together. Each SNP was coded as 0, 1, or 2 copies of the risk allele except for the SNP rs5934683 in chromosome X that was coded 0, 0.5, and 1. We defined the GRS as the count of risk alleles across all 21 SNPs, ranging from 12 to 33. Since the published effects of each SNP were similar, an unweighted GRS was preferred. We also explored the models using weights derived from the GWAS publications and models fitted to our data, but the predictive accuracy was very similar.

The predictive accuracy of models was assessed with the area under the ROC curve (AUROC), adjusted for the propensity score. Data were split into quintiles of propensity scores, and the weighted mean of the AUROC for each quintile model was calculated. Weights were proportional to the number of cases in each quintile. To account for potential overfitting that could overestimate the effect of GRS, especially for more complex models using weights, 5-fold cross validation was used to estimate the AUROC. In addition, the 95% CIs were calculated using bootstrapping techniques on top of the cross-validated estimates.

To estimate the potential public health impact of the ERS and GRS, we applied the estimated odds ratios (OR) to population average CRC incidence estimations published by the International Agency for Research on Cancer (IARC). Data were extracted from the publication Cancer Incidence in Five Continents (CI5) Volume X, for the Spanish cancer registries^24^. Average age and gender-specific cumulative risks for the Spanish population were projected according to combinations of ERS and GRS to define risk strata. For these estimates, the published cumulative risks were multiplied by the ORs estimated from out risk models. We used the average number of risk factors and risk alleles in the population as reference categories for these calculations. Also, the sensitivity and specificity values for a selection of risk scores were used, combined with the cumulative risk of developing CRC cancer for age decades from 40 years to 80 years old, in order to estimate the positive and negative predictive values. The Bayes theorem was used for these calculations.

Statistical analysis was carried out using R statistical software (R Foundation for Statistical Computing, Vienna, Austria).

## Results

**Figure 1:**
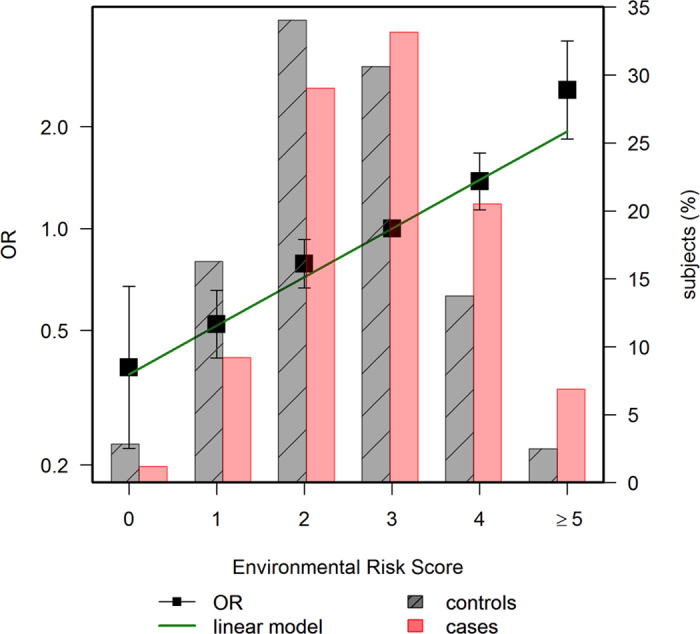
Distribution and CRC risk of the environmental risk score in cases and controls. The left axis scale indicates the OR for CRC according to the number of environmental risk factors. The category of tree factors was selected as reference (OR = 1), because this is the average in the population. The right axis scale indicates the proportion of cases and controls shown in bars for each number of environmental risk factors.

Case and control characteristics are detailed in Table 2. Variables were coded with the lower CRC risk category as reference to simplify the effects of comparison and calculation of risk scores. All the environmental variables considered for the risk model were significantly associated with CRC, after adjusting for the propensity score. The crude ORs were very similar for the categorizations selected, ranging from 1.29 (BMI ≥30 mg/kg^2^) to 1.57 (NSAID/ASA). The multivariate model with all environmental factors showed that all were independently contributing to CRC risk (Table 3). Tobacco was not included in the model since smoking was no longer significant when other factors were considered (adjusted OR 1.06, 95% CI 0.91 to 1.23). The ERS, calculated as the count of risk factors, indicated that on average the adjusted OR was 1.36 (95% CI 1.27 to 1.45). Figure 1 shows the distribution of the ERS for cases and controls, and the estimated risk of CRC according to the number of risk factors, compared to an average individual (ERS = 3).

Family history of CRC was strongly associated with CRC (adjusted OR 2.27, 95% CI 1.88 to 2.74). We combined first, second, or third-degree relatives with CRC in the risk group, since the ORs were very similar. This variable was independent of the environmental risk factors.

**Figure 2:**
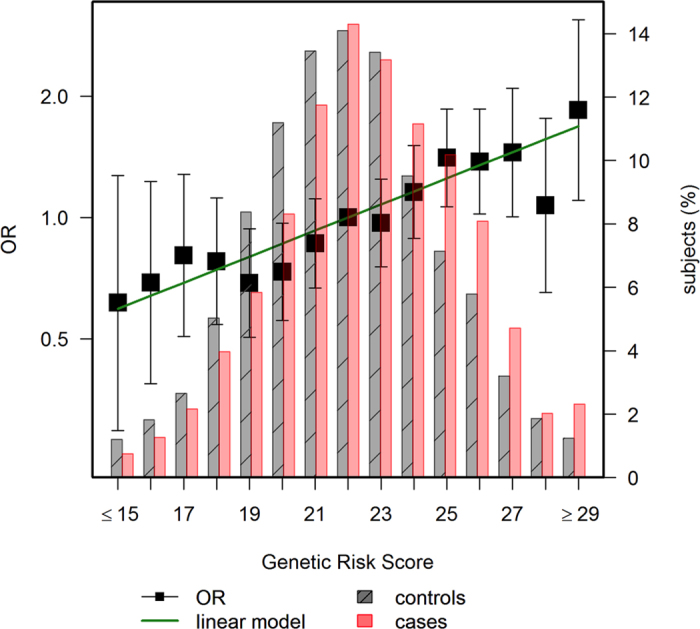
Distribution and CRC risk of the genetic risk score in cases and controls. The left axis scale indicates the OR for CRC according to the number of risk alleles. The group of 22 alleles was selected as reference category (OR = 1), because this is the average in the population. The right axis scale indicates the proportion of cases and controls shown in bars for each allele.

Out of 21 GWAS SNPs analysed, only 5 were statistically significant in our data (rs10752881, rs6983267, rs9929218, rs4939827, rs961253; Table 1). The contribution to risk of each SNP in the MCC-Spain study was small, with per allele ORs in the range of 1.00 to 1.22. The GRS built as the unweighted count of risk alleles was significantly associated with CRC, with an average per-allele OR of 1.07 (95% CI 1.04 to 1.10). The GRS was significantly associated with family history, but it only explained 0.3% of the variability. Subjects with family history had an average of 0.45 (95% CI 1.18 to 1.78, *p* = 0.0004) more risk alleles, and four SNPs (rs16892766, rs10795668, rs9929217, and rs4939827) were associated with family history of CRC with *p*-value < 0.05. When the GRS was adjusted for family history of CRC, the OR was 1.07 (95% CI 1.05 to 1.10, *p* = 1.2e-8). Cases had an average of 22.73 alleles while controls had 22.10, with ample overlap as shown in the histogram of Fig. 2. The difference in mean GRS was 0.63 alleles (95% CI 0.44 to 0.82; *p* = 1.2e-10). There was an 82% increase in CRC risk (OR 1.82, 95% CI 1.11 to 2.98) between subjects with ≤18 risk alleles (first quintile) and those with ≥26 risk alleles (fifth quintile). As shown in Fig. 2, the increase in risk per allele was linear, indicating the independent additive contribution of each allele to the GRS. The risk of CRC doubled for a difference of 10 risk alleles (OR 1.96, 95%CI 1.54 to 2.50). The GRS was independent of environmental variables. Also, no significant interactions were observed between the GRS and age, sex, or any of the environmental variables included in the multivariate model.

Regarding tumour location, there were 30.7% (n = 405) tumours located in the rectum, 40.2% (n = 531) in the distal colon, and 29.1% (n = 385) in the proximal colon (15 subjects had missing data). The analysis stratified by cancer location did not show relevant differences (Supplementary Table 1). In general, both environmental and genetic factors had greater effects in rectal than colon cancer. High intake of red meat was the factor with major differences between colon and rectal cancers.

### Predictive accuracy of the risk model

**Figure 3:**
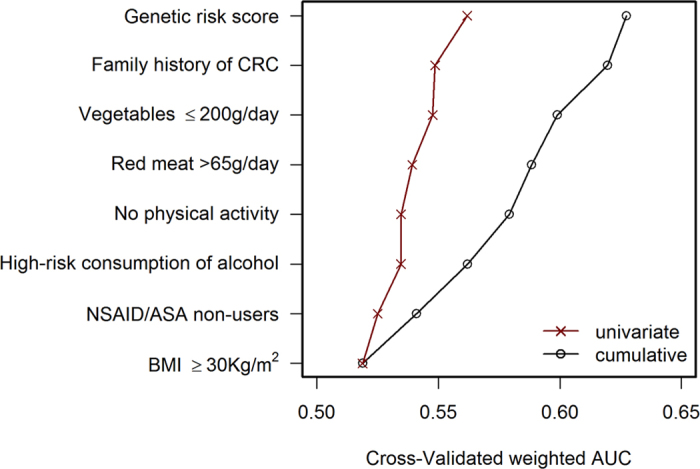
Individual and cumulative contribution of each factor to CRC predictive accuracy. The area under the ROC curve (AUROC), as indicator of predictive accuracy for each variable in the risk model, is shown. The left discontinuous (red) line indicates the individual contribution of each variable, and the right continuous (black) line indicates the cumulative contribution, bottom to top. Environmental variables are sorted by increasing AUROC. CRC: colorectal cancer; NSAID: nonsteroidal anti-inflammatory drugs; ASA: acetylsalicylic acid; BMI: body mass index.

The contribution to CRC risk prediction was estimated for modifiable environmental risk factors, family history, and the GRS. Figure 3 shows the individual (red discontinuous line) and cumulative (black continuous line) contribution of each environmental factor to the risk. The cumulative contribution of the seven environmental factors resulted in a cross-validated AUROC of 0.60 (95% CI 0.57 to 0.61). Family history, which is not modifiable but can be obtained by interview, increased the AUROC to 0.61 (95% CI 0.59 to 0.64). The GRS, on its own, had an AUROC of 0.56 (95% CI 0.54 to 0.58). The increase in AUROC for the model with the GRS on top of ERS and family history (FH) was 0.02, with an overall AUROC of 0.63 (95% CI 0.60 to 0.66). This 5-fold cross-validated AUROC was smaller than the direct estimate of the model, which was 0.65, indicating some optimism in the estimate even when an unweighted GRS was used. When we explored weighted models for the GRS, the 5-fold cross-validated AUROC was 0.62 (95%CI 0.60 to 0.65) for weights derived from published GWAS and 0.63 (95% CI 0.61 to 0.66) for weights derived from the fitted logistic regression model.

### Estimating the potential public health impact of a risk model to stratify screening or modify risk factors in the Spanish average risk population

A simple calculation of the relative risk could be made using the following risk score (RS) equation: RS = 1.36^(ERS-3)^ * 2.25^FH^ * 1.07^(GRS-22)^. An individual with no family history (FH = 0), three environmental risk factors (ERS = 3), and 22 risk alleles (GRS = 22) would have the average population risk (RS = 1). In contrast, a subject with 6 environmental risk factors (ERS = 6), family history (FH = 1), and 28 risk alleles (GRS = 28) would have an RS of 7.25 times the average risk. The distribution in the population of the RS is shown in Supplementary Figure 1.

**Figure 4:**
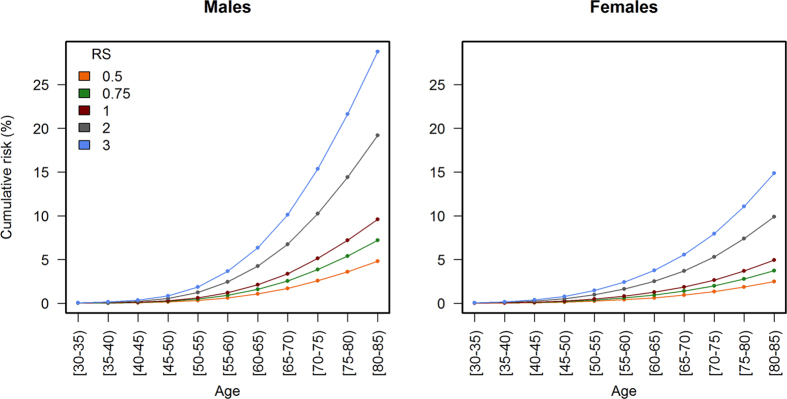
Estimation of CRC incidence in Spain by sex, age (years), and risk score. Color lines indicate age-specific cumulative risk rates of CRC per 100 individuals in Spain according to sex and risk score (RS), for a selection of values. The cumulative risk curve for the average individual corresponds to RS = 1. The risk score can be calculated as RS = 1.36^(ERS-3)^ * 2.25^FH^ * 1.07^(GRS-22)^, where ERS is the number of environmental risk factors (average 3 in the population), FH is the presence of family history of CRC (0 = no, 1 = yes), and GRS is the number of risk alleles (average 22 in the population).

As lifetime cumulative risk is a better individual measure of the impact of cancer burden, we calculated individual risk by applying the estimated RS to specific cumulative risk of CRC of the Spanish population. For this calculation, Spanish cancer incidence data obtained from cancer registries were used. Figure 4 shows how cumulative incidence curves are shifted according to the risk score. Supplementary Figures 2 and 3 show these analyses but specific to the ERS and GRS, respectively. As it is already known, men have a higher incidence of CRC than women, and incidence grows exponentially from 50 years of age for both sexes.

From Fig. 4 (numbers are shown in Supplementary Table 2) we can estimate that a Spanish man with average risk score (RS = 1, 22 risk alleles) has a lifetime cumulative risk of CRC of approximately 10% (5% in the case of a woman). In contrast, the lifetime cumulative risk would increase to 20% and 10% for men and women, respectively, among subjects with a risk score of 2 (29 risk alleles). The risk for a hypothetical individual at age 50 with an RS of 2 is similar as that for an individual with average risk alleles (GRS = 22) but younger (45 years old for men). In other words, at age 45, this man with RS = 2 would be at the same risk as a man with RS = 1 at age 50. At older ages, since the effect is multiplicative, the relative risk anticipation is greater. The cumulative risk of CRC during the screening age period (50–69), in this scenario, would double: from 3% to 6% among men and from 2% to 4% for women.

**Figure 5:**
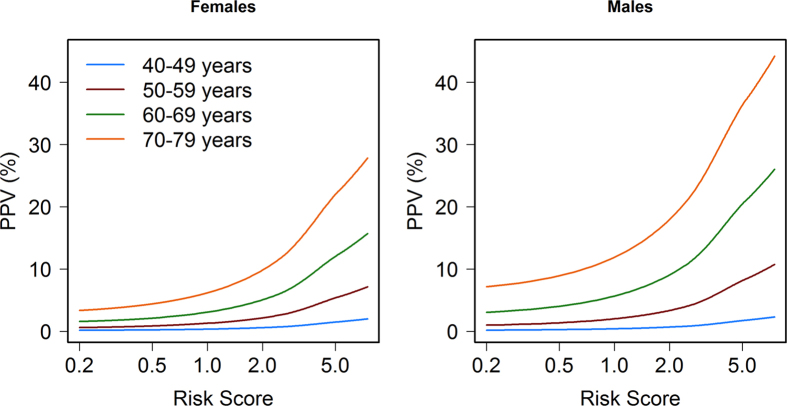
Positive predictive value for CRC according to age range and risk score. Colour lines indicate the positive predictive value (PPV) for CRC for each age range. Estimates are derived from sensitivity and specificity of the risk model (Table 4) for each risk score applied to the cumulative risk of developing CRC in the age range, using Bayes’ theorem.

The sensitivity, specificity, and likelihood ratios of the risk model to detect CRC for selected risk score cut-offs are shown in Table 4. The use of a high cut-off (RS = 5) offers high specificity (98.94%) but low sensitivity (8.38%). These figures can be useful to assess the relative interest of extending the age of CRC screening for selected strata of the population with such high risk scores, either before age 50 or after age 69. As Fig. 5 and Supplementary Table 3 show, the positive predictive value of the model increases only in a relevant way at older ages, when the prior probability of CRC is higher, especially for RS over 2. The cumulative risk of developing CRC during the age range 70–79 is almost 40% for subjects with a risk score of 5.

## Discussion

We assessed the potential utility of a risk prediction model for CRC that combines modifiable risk factors with family history of CRC and a genetic risk score based on 21 susceptibility SNPs. We have observed that modifiable risk factors have a stronger value for risk prediction than does genetic susceptibility. Though the added value of each SNP is small, the combination of 21 SNPs adds significantly to the predictive power of the risk model.

Our study is large enough to confirm that established risk factors are associated with risk: family history of CRC, high consumption of alcohol, obesity, lack of physical activity in leisure time, high intake of red meat, low intake of vegetables, and non-use of NSAIDs/ASA. These risk factors were selected based on previous evidence reported in systematic reviews and meta-analyses^25^,^26^,^27^,^28^,^29^,^30^,^31^. All were independent predictors of CRC in an average risk population, with the exception of smoking, which was only significant in the univariate analysis. A recent meta-analysis on smoking has shown that the effect is small for CRC, with a summary OR smaller than 1.25, and larger for rectal than colon cancer^32^. We also analysed other covariates that have been associated with CRC (diabetes mellitus, inflammatory bowel disease, and diverticulitis) but they were not associated with CRC in our study, perhaps because of the small number of affected individuals. Nor was intake of vitamin D, calcium, or folic acid associated with CRC. We opted not to include statins in the model since there is controversy regarding these drugs and CRC risk^33^.

Our study confirms that family history of CRC is the strongest single risk factor for CRC. We found a significant association between the GRS and family history, which highlights the importance of genetic susceptibility in CRC, though family history could also contribute to risk through shared lifestyle or environmental factors. Also, gene-environment interactions may play a role in this type of cancer^34^,^35^,^36^.

Our analysis has shown that the ERS, built as an additive model of modifiable factors, has stronger association with CRC than the GRS. On average, each environmental risk factor increases CRC risk by 35%, while each risk allele only increases it by 7%. This implies that the change of one modifiable risk factor towards healthier lifestyle might offset the effect of 4 risk alleles. Given the fact that environmental factors explain a significant part of the CRC risk, we believe it to be important to give thought to incorporating clinical data to improve current screening and encourage patients to achieve a healthier lifestyle.

We also believe it is important to consider that our genotyping array only had 21 susceptibility SNPs, and today more than 60 have been identified in diverse GWAS studies^22^. Though SNPs identified more recently have smaller effects (in the range of 5% increased risk per allele) and smaller allele frequencies, their addition may still increase the predictive accuracy of the model in a relevant way. In our Spanish population only five SNPs out of the 21 analysed were significantly associated with CRC risk. This might be related to lack of statistical power, since with 1300 cases and 2700 controls we only have 30% power to detect an OR of 1.10, but some of the SNPs may also have effects limited to specific populations. It is reassuring, however, that all SNPs analysed had an effect in the same direction as reported in the discovery study.

Several risk prediction models for advanced neoplasia or CRC have previously been published, with AUROC between 0.65 and 0.75^8^. Our estimate of predictive accuracy, corrected for overfitting through cross-validation, is slightly smaller (AUROC: 0.63, 95% CI 0.60 to 0.66), but our model could not include age and gender because these factors were used to match the controls. Also the estimated risk per allele (OR 1.07, 95% CI 1.04 to 1.10) was similar to those reported by studies which included genetic biomarkers and phenotypic variables (AUROC: 0.51–0.73)^6^,^11^,^12^,^13^,^14^. Dunlop *et al*.^12^ used the combined effect of age, gender, family history, and 10 SNPs to assess CRC risk. They reported an AUROC of 0.59 and a mean per-allele increase in risk of 9%. Yarnall *et al*.^11^ combined alcohol intake with 14 SNP obtaining an AUROC of 0.61. Hsu *et al*.^6^ developed a risk determination model based on family history and 27 SNPs with an AUC of 0.56 and a risk per allele of 1.02–1.12. Finally, Jung *et al*.^14^ reported an AUC of 0.74 with a model that included age, sex, smoking status, fasting serum glucose, family history of colorectal cancer, and 23 SNP.

Our study, which used more SNPs than most previous studies, as well as questionnaire data including diet, confirms that the AUROC increases with more SNPs. Furthermore, the aim of our study was also to build a risk model useful to tailoring CRC screening programs according to individuals’ characteristics and calculating the potential impact of determining an individual risk score in a CRC screening population. The risk model, applied to Spanish cancer registry cumulative risk of CRC, has shown that 3 modifiable risk factors or 10 risk alleles have an expected advance of 5 years in the incidence curve of men by age 50 (2.5 years for women). The absolute effect on incidence is larger at older ages, since the effect is multiplicative. This implies that screening in average-risk populations probably should start earlier, at 45 years, for individuals with more risk factors, and could be delayed to 55 years old (or 60) for individuals with fewer risk factors or risk alleles.

Our calculations also show that it would be most useful to extend the age of CRC screening for high risk population after age 69. The positive predictive value of the model increases significantly at older ages, when the prior probability of CRC is higher. Since the conditional life expectancy of a person at age 70 still is long, extending the screening until age 79 might yield a greater reduction in CRC burden.

Moreover, we could also use the risk model to select high-risk subjects in whom colonoscopy might be the optimal initial screening technique rather than the less sensitive faecal occult blood test that is currently implemented in Spain and many other countries in Europe^37^. Another important point to highlight is that the use of prediction models, together with good communication tools, could increase the individual perceived risk, and consequently the participation rate and adherence to screening, especially in high-risk subjects. Moreover, the awareness of a personal risk of CRC might improve people’s lifestyle and thereby reduce CRC incidence.

This study has some limitations. Our model was developed within a retrospective case-control setting, and relied on self-reported data. So, measurement error and recall bias may have led to an underestimation of the predictive accuracy. Cases and controls were not well matched regarding age, sex, and education. However, we performed all the analyses adjusted for a propensity score to reduce the possible bias related to this problem. The model is only applicable to asymptomatic individuals from the general population (average risk); subjects with symptoms or several affected relatives should be referred to colonoscopy independently of the risk score.

As already mentioned, this study only included 21 risk SNPs, while more than sixty have already been identified. More studies are needed to determine the generalizability, usefulness of information, and the cost-effectiveness of applying individual genotyping in a CRC screening program. However, it should be noted that the cost of whole-genome genotyping is decreasing, its determination only needs to be performed once in a lifetime, and the data probably will be useful for predicting risk of other diseases in addition to cancer.

In conclusion, we assessed the predictive accuracy of a model for CRC that could be useful to stratify the population into risk categories and tailor CRC screening by adapting the onset age, the intensity, and the screening test. In our model, although the genetic factors are significant contributors, the modifiable risk factors contribute more significantly. Risk assessment may increase screening participation and adoption of healthier lifestyles.

## Additional Information

**How to cite this article:** Ibáñez-Sanz, G. *et al*. Risk Model for Colorectal Cancer in Spanish Population Using Environmental and Genetic Factors: Results from the MCC-Spain study. *Sci. Rep.* **7**, 43263; doi: 10.1038/srep43263 (2017).

**Publisher's note:** Springer Nature remains neutral with regard to jurisdictional claims in published maps and institutional affiliations.

## Supplementary Material

Supplementary Tables and Figures

## Figures and Tables

**Table 1:** Association between the 21 selected previously reported SNPs and risk of CRC in the study population.

SNP	Chr	Position	Mapped Gene	Risk Allele	Risk Allele Frequency	Reported *p*-value	Reported OR	OR MCC-Spain	95% CI	*p*-value
**rs10752881**	**1**	**183004356**	**KRT18P28 - LAMC1**	**A**	**0.76**	**5.0E-06**	**1.07**	**1.11**	**1.01–1.21**	**0.04**
**rs6691170**	1	221872104	DUSP10 - QRSL1P2	T	0.31	1.0E-09	1.06	1.09	0.99–1.20	0.08
**rs10936599**	3	169774313	MYNN	C	0.19	3.0E-08	1.04	1.10	0.98–1.23	0.11
**rs1321311**	6	36655123	N/A	C	0.30	1.0E-10	1.10	1.03	0.93–1.15	0.54
**rs7758229**	6	160419220	SLC22A3	T	0.67	8.0E-09	1.28	1.07	0.96–1.18	0.21
**rs16892766**	8	116618444	LINC00536 - EIF3H	C	0.39	3.0E-18	1.27	1.17	0.98–1.39	0.08
**rs6983267**	**8**	**127401060**	**CCAT2 - LOC101930033**	**G**	**0.82**	**1.0E-14**	**1.27**	**1.10**	**1.00–1.21**	**0.04**
**rs10795668**	10	8659256	RNA5SP299 - LINC00709	G	0.73	5.0E-15	1.15	1.06	0.95–1.17	0.30
**rs4948317**	10	58811675	BICC1	C	0.30	7.0E-08	1.10	1.07	0.97–1.18	0.19
**rs3802842**	11	111300984	COLCA2 - COLCA1	C	0.08	6.0E-10	1.11	1.09	0.98–1.20	0.12
**rs3824999**	11	74634505	POLD3	G	0.13	4.0E-10	1.08	1.09	1.00–1.20	0.06
**rs10879357**	12	72020783	TPH2	G	0.50	3.0E-06	1.25	1.00	0.91–1.11	0.94
**rs11169552**	12	50761880	DIP2B - ATF1	C	0.82	2.0E-10	1.09	1.02	0.91–1.14	0.80
**rs7315438**	12	115453598	TBX3 - UBA52P7	T	0.41	6.0E-06	1.11	1.03	0.94–1.13	0.53
**rs4444235**	14	53944201	RPS3AP46 - MIR5580	C	0.43	8.0E-10	1.11	1.03	0.94–1.13	0.51
**rs9929218**	**16**	**68787043**	**CDH1**	**G**	**0.46**	**1.0E-08**	**1.10**	**1.13**	**1.01–1.25**	**0.03**
**rs4939827**	**18**	**48927093**	**SMAD7**	**T**	**0.27**	**8.0E-28**	**1.20**	**1.22**	**1.11–1.34**	**0.03**
**rs10411210**	19	33041394	RHPN2	C	0.31	5.0E-09	1.15	1.01	0.88–1.16	0.92
**rs4925386**	20	62345988	LAMA5	C	0.58	2.0E-10	1.08	1.08	0.98–1.19	0.14
**rs961253**	**20**	**6423634**	**FGFR3P3 - CASC20**	**A**	**0.28**	**2.0E-10**	**1.12**	**1.10**	**1.00–1.22**	**0.05**
**rs5934683**	X	9783434	GPR143 - SHROOM2	C	0.57	7.0E-10	1.07	1.04	0.93–1.17	0.46

SNPs associated with CRC risk in MCC population with *p* < 0.05 are highlighted in bold.

**Table 2:** Characteristics of the MCC-Spain study participants.

Characteristic	Control		Case		Crude OR	95% CI
	n	%	n	%		
Age						
25–50 years	394	14.43	80	6.04	1.00	
50–70 years	1441	52.76	649	48.98	2.22	1.71–2.87
70–90 years	909	33.28	607	45.81	3.29	2.53–4.27
Sex						
Female	1275	46.47	471	35.25	1.00	
Male	1469	53.53	865	64.75	1.59	1.39–1.82
Family History of CRC						
No	2411	87.86	1044	78.14	1.00	
Yes	333	12.14	292	21.86	2.25	1.87–2.71
Smoking						
Non-smoker	1195	43.55	557	41.69	1.00	
Former/Current smoker	1549	56.45	779	58.31	1.20	1.04–1.38
Alcohol						
Low consumption	2317	84.44	1036	77.54	1.00	
High consumption	427	15.56	300	22.46	1.38	1.16–1.63
Body Mass Index at age 45						
<30 kg/m^2^	2556	93.15	1194	89.37	1.00	
≥30 kg/m^2^	188	6.85	142	10.63	1.36	1.07–1.73
Physical activity in leisure time (MET)						
Yes	1687	61.48	717	53.67	1.00	
No	1057	38.52	619	46.33	1.37	1.19–1.58
Vegetables						
>200 g/day	846	30.83	345	25.82	1.00	
≤200 g/day	1898	69.17	991	74.18	1.39	1.19–1.62
Red meat						
≤65 g/day	1621	59.07	662	49.55	1.00	
>65 g/day	1123	40.93	674	50.45	1.38	1.20–1.59
NSAID/ASA						
Regular use in the last year	1995	72.70	1064	79.64	1.00	
Non-use/sporadically use	749	27.30	272	20.36	1.54	1.31–1.82

MET: Metabolic equivalent of task per hour per week; NSAID: Nonsteroidal anti-inflammatory drugs; ASA: acetylsalicylic acid.

**Table 3:** Multivariate-adjusted risk factors associated with CRC.

		Adjusted OR^a^	CI 95%
Genetic Risk Score	GRS (per allele)	1.07	1.04–1.10
Family history of CRC		2.25	1.87–2.72
Environmental risk factors	Alcohol	1.34	1.12–1.60
	BMI ≥ 30 kg/m^2^	1.29	1.01–1.65
	No physical activity	1.34	1.16–1.55
	Vegetables ≤ 200 g/day	1.36	1.15–1.58
	Red meat > 65 g/day	1.29	1.12–1.49
	No NSAID/ASA regular use	1.57	1.33–1.86
	ERS (per factor)	1.36	1.27–1.45

CRC: colorectal cancer; GRS: genetic risk score; ERS: environmental risk score; BMI: body mass index; NSAID: nonsteroidal anti-inflammatory drugs; ASA: acetylsalicylic acid.

^a^All variables are adjusted by propensity score and all the variables shown in the table.

^b^The reference category is 22 risk alleles, the average in the population.

**Table 4:** Predictive performance indexes of the risk score for selected cutoffs.

Risk score	Sensitivity	Specificity	Positive Likelihood Ratio	Negative Likelihood Ratio
0.25	98.50	7.87	1.07	0.19
0.5	91.39	30.72	1.32	0.28
1	71.48	60.13	1.79	0.47
2	41.62	84.66	2.71	0.69
4	13.55	97.89	6.41	0.88
5	8.38	98.94	7.93	0.93
6	5.39	99.31	7.78	0.95
