# Corrigendum: Risk Model for Colorectal Cancer in Spanish Population Using Environmental and Genetic Factors: Results from the MCC-Spain study

**DOI:** 10.1038/srep46807

**Published:** 2017-05-17

**Authors:** Gemma Ibáñez-Sanz, Anna Díez-Villanueva, M. Henar Alonso, Francisco Rodríguez-Moranta, Beatriz Pérez-Gómez, Mariona Bustamante, Vicente Martin, Javier Llorca, Pilar Amiano, Eva Ardanaz, Adonina Tardón, Jose J. Jiménez-Moleón, Rosana Peiró, Juan Alguacil, Carmen Navarro, Elisabet Guinó, Gemma Binefa, Pablo Fernández-Navarro, Anna Espinosa, Verónica Dávila-Batista, Antonio José Molina, Camilo Palazuelos, Gemma Castaño-Vinyals, Nuria Aragonés, Manolis Kogevinas, Marina Pollán, Victor Moreno

Scientific Reports 7: Article number: 4326310.1038/srep43263; published online: 02 24 2017; updated: 05 17 2017

The original version of this Article contained a typographical error in the spelling of the author Pablo Fernández-Navarro, which was incorrectly given as Pablo Fernández Navarro. This has now been corrected in both the PDF and HTML versions of the Article.

