# Supplementary material for: Risk Model for Colorectal Cancer in Spanish Population Using Environmental and Genetic Factors: Results from the MCC-Spain study
Source: Sci Rep. 2017 Feb 24;7:43263. doi: 10.1038/srep43263 (PMC5324108; doi:10.1038/srep43263)
Supplement: Supplementary Tables and Figures [file srep43263-s1.doc]

**Supplementary tables and figures**

**Risk Model for Colorectal Cancer in Spanish Population Using Environmental and Genetic Factors. Results from the MCC-Spain study.**

Gemma Ibáñez-Sanz; Anna Díez-Villanueva; M. Henar Alonso; Francisco Rodríguez-Moranta; Beatriz Pérez-Gómez; Mariona Bustamante; Vicente Martin; Javier Llorca; Pilar Amiano; Eva Ardanaz; Adonina Tardón; Jose J. Jimenez-Monleon; Rosana Peiro; Juan Alguacil; Carmen Navarro; Elisabet Guinó; Gemma Binefa; Pablo Fernández Navarro; Anna Espinosa; Verónica Dávila-Batista; Antonio José Molina; Camilo Palazuelos; Gemma Castaño-Vinyals; Nuria Aragonés; Manolis Kogevinas; Marina Pollan; Victor Moreno* .

**Supplementary Table 1.** Analyses of predictive factors associated with the risk of colon cancer and rectal cancer

|  | **Risk of Colon Cancer** | | **Risk of Rectal Cancer** | |  | **Rectal vs Colon** | |
| --- | --- | --- | --- | --- | --- | --- | --- |
|  | **OR^a^** | **CI 95%** | **OR^a^** | **CI 95%** |  | **OR^a^** | **CI 95%** |
| **Genetic Risk Score** | **1.06** | **1.03-1.09** | **1.10** | **1.06-1.15** |  | 1.04 | 1.00-1.08 |
| **Family History of CRC** | **2.27** | **1.85-2.80** | **2.23** | **1.68-2.95** |  | 1.01 | 0.76-1.35 |
| **Smoking** | 1.00 | 0.85-1.18 | 1.23 | 0.97-1.56 |  | 1.28 | 1.00-1.65 |
| **Alcohol** | **1.30** | **1.06-1.59** | **1.38** | **1.05-1.81** |  | 1.01 | 0.76-1.36 |
| **BMI ≥30kg/m^2^** | 1.28 | 0.97-1.69 | 1.28 | 0.88-1.85 |  | 1.02 | 0.70-1.49 |
| **No Physical Activity** | **1.34** | **1.14-1.57** | **1.38** | **1.11-1.73** |  | 1.01 | 0.79-1.28 |
| **Vegetables ≤200g/day** | **1.29** | **1.09-1.52** | **1.63** | **1.28-2.08** |  | 1.29 | 1.00-1.67 |
| **Red Meat >65g/day** | 1.14 | 0.96-1.36 | **1.61** | **1.28-2.02** |  | **1.46** | **1.14-1.87** |
| **No NSAID/ASA Regular Use** | **1.59** | **1.31-1.92** | **1.64** | **1.25-2.15** |  | 1.03 | 0.77-1.38 |

Associations with *p*<0.05 are shown in bold.

^a^Each variable is adjusted by the propensity score and the other variables in the table.

CRC: colorectal cancer; BMI: body mass index; NSAID: nonsteroidal anti-inflammatory drugs; ASA: acetylsalicylic acid.

**Supplementary Figure 1.** Distribution of the risk score in the MCC-Spain study population


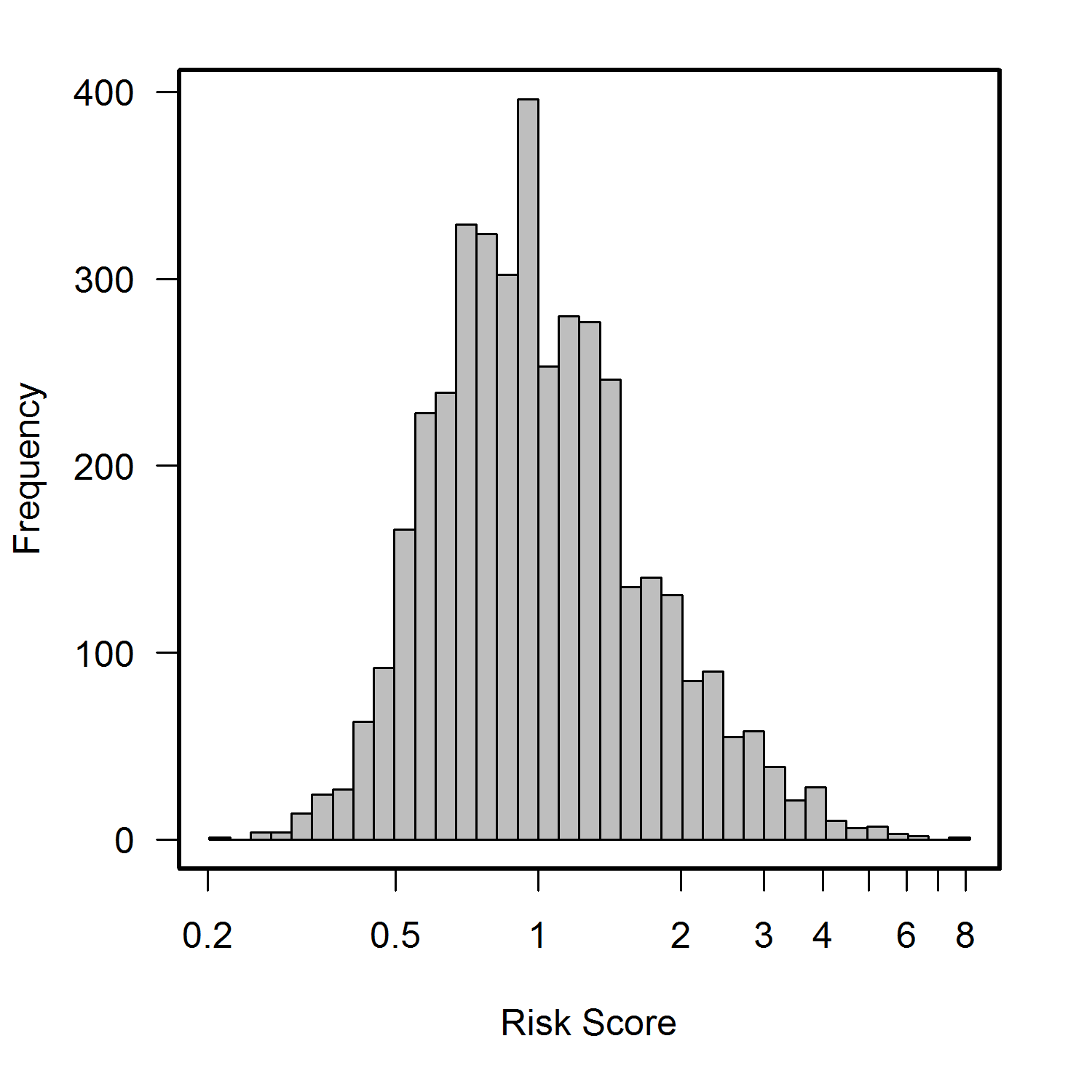


**Supplementary Figure 2.** Estimation of CRC incidence in Spain by sex, age (years) and
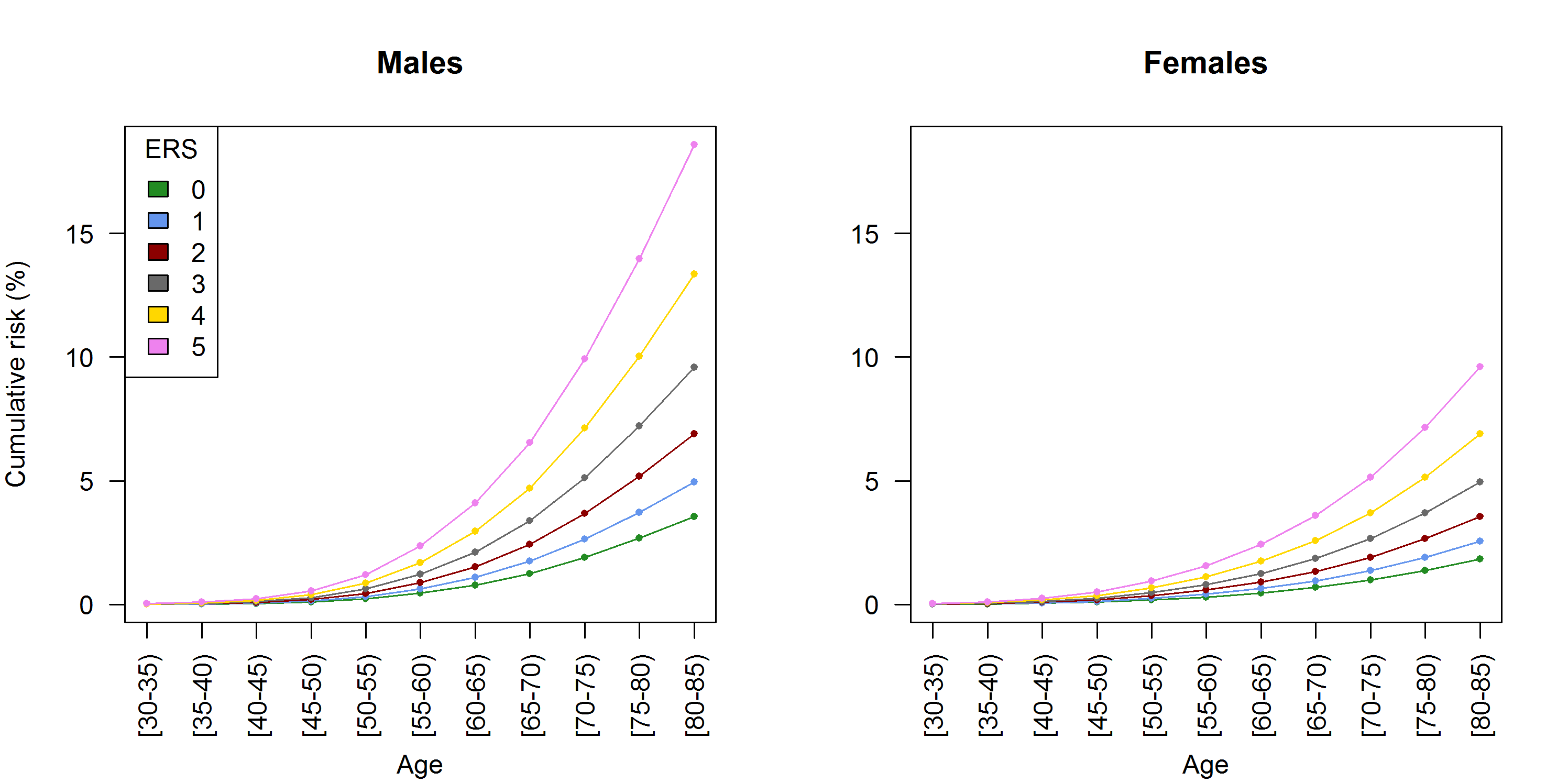
environmental risk score (ERS).

**Supplementary Figure 3.** Estimation of CRC incidence in Spain by sex, age (years) and
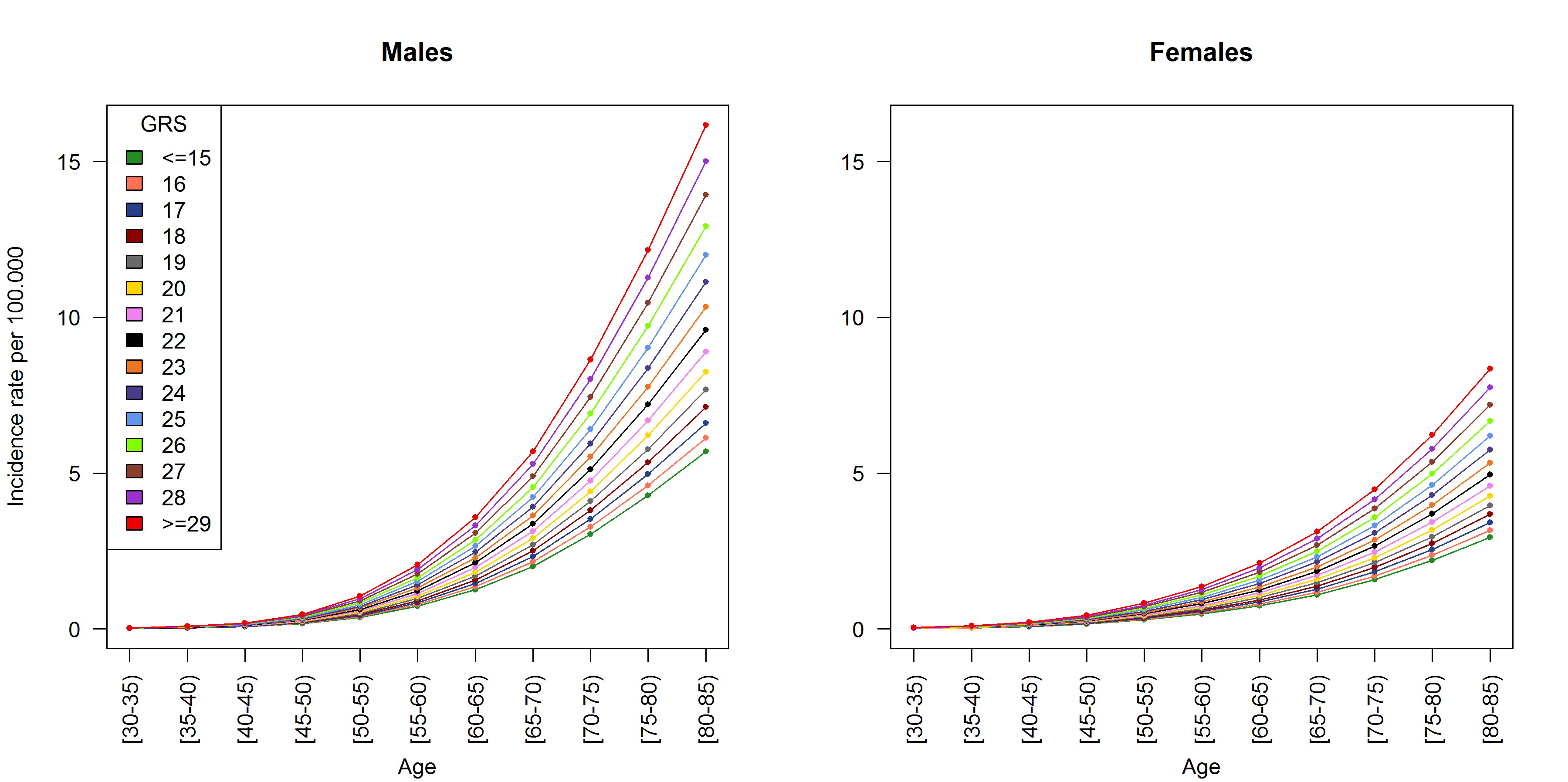
genetic risk score (GRS).

**Supplementary Table 2.** Cumulative risk of CRC according to age range and risk score (males). Numbers for figure 4.

|  | **Risk score** | | | | | | | | | | | |
| --- | --- | --- | --- | --- | --- | --- | --- | --- | --- | --- | --- | --- |
|  | **Males** | | | | |  | **females** | | | | | |
| **Age (years)** | **0.5** | **0.75** | **1** | **2** | **3** |  | **0.5** | **0.75** | **1** | **2** | **3** |  |
| 15-20 | 0 | 0 | 0 | 0 | 0 |  | 0 | 0 | 0 | 0 | 0 |  |
| 20-25 | 0 | 0 | 0 | 0.01 | 0.01 |  | 0 | 0 | 0 | 0.01 | 0.01 |  |
| 25-30 | 0 | 0.01 | 0.01 | 0.02 | 0.02 |  | 0 | 0.01 | 0.01 | 0.02 | 0.03 |  |
| 30-35 | 0.01 | 0.01 | 0.02 | 0.04 | 0.05 |  | 0.01 | 0.02 | 0.02 | 0.04 | 0.07 |  |
| 35-40 | 0.02 | 0.03 | 0.05 | 0.09 | 0.14 |  | 0.03 | 0.04 | 0.05 | 0.11 | 0.16 |  |
| 40-45 | 0.06 | 0.08 | 0.11 | 0.22 | 0.34 |  | 0.06 | 0.10 | 0.13 | 0.25 | 0.38 |  |
| 45-50 | 0.14 | 0.21 | 0.28 | 0.56 | 0.83 |  | 0.13 | 0.19 | 0.25 | 0.51 | 0.76 |  |
| 50-55 | 0.31 | 0.46 | 0.62 | 1.24 | 1.86 |  | 0.24 | 0.37 | 0.49 | 0.97 | 1.46 |  |
| 55-60 | 0.61 | 0.91 | 1.22 | 2.43 | 3.65 |  | 0.40 | 0.60 | 0.81 | 1.61 | 2.42 |  |
| 60-65 | 1.06 | 1.59 | 2.12 | 4.24 | 6.36 |  | 0.63 | 0.94 | 1.25 | 2.50 | 3.75 |  |
| 65-70 | 1.69 | 2.53 | 3.38 | 6.75 | 10.13 |  | 0.92 | 1.39 | 1.85 | 3.70 | 5.55 |  |
| 70-75 | 2.56 | 3.84 | 5.12 | 10.25 | 15.37 |  | 1.33 | 1.99 | 2.65 | 5.31 | 7.96 |  |
| 75-80 | 3.60 | 5.41 | 7.21 | 14.41 | 21.62 |  | 1.85 | 2.77 | 3.69 | 7.39 | 11.08 |  |
| 80-85 | 4.80 | 7.19 | 9.59 | 19.18 | 28.77 |  | 2.48 | 3.71 | 4.95 | 9.90 | 14.85 |  |

**Supplementary Table 3.** Positive predictive value for CRC according to age range and risk score. Numbers for Figure 4.

|  |  | **Positive predictive values** | | | |
| --- | --- | --- | --- | --- | --- |
|  |  |  |  |  |  |
|  | **Risk score** | **40-49 years** | **50-59 years** | **60-69 years** | **70-79 years** |
| **Females** | **0.2** | 0.20 | 0.77 | 1.85 | 3.74 |
|  | **0.3** | 0.22 | 0.84 | 2.00 | 4.05 |
|  | **0.4** | 0.24 | 0.92 | 2.18 | 4.41 |
|  | **0.5** | 0.26 | 0.99 | 2.36 | 4.75 |
|  | **0.6** | 0.28 | 1.06 | 2.51 | 5.06 |
|  | **0.7** | 0.30 | 1.13 | 2.69 | 5.40 |
|  | **0.8** | 0.32 | 1.21 | 2.87 | 5.76 |
|  | **0.9** | 0.34 | 1.28 | 3.04 | 6.07 |
|  | **1.0** | 0.36 | 1.34 | 3.17 | 6.34 |
|  | **2.0** | 0.54 | 2.01 | 4.73 | 9.30 |
|  | **3.0** | 0.78 | 2.90 | 6.72 | 12.96 |
|  | **4.0** | 1.26 | 4.63 | 10.49 | 19.49 |
|  | **5.0** | 1.56 | 5.67 | 12.67 | 23.06 |
|  | **6.0** | 1.53 | 5.57 | 12.46 | 22.72 |
|  | **7.0** | 1.68 | 6.09 | 13.53 | 24.43 |
| **Males** | **0.2** | 0.24 | 1.20 | 3.43 | 7.36 |
|  | **0.3** | 0.26 | 1.30 | 3.70 | 7.93 |
|  | **0.4** | 0.28 | 1.42 | 4.04 | 8.61 |
|  | **0.5** | 0.31 | 1.54 | 4.35 | 9.24 |
|  | **0.6** | 0.33 | 1.64 | 4.63 | 9.81 |
|  | **0.7** | 0.35 | 1.76 | 4.95 | 10.45 |
|  | **0.8** | 0.37 | 1.88 | 5.28 | 11.10 |
|  | **0.9** | 0.40 | 1.99 | 5.57 | 11.67 |
|  | **1.0** | 0.41 | 2.08 | 5.82 | 12.15 |
|  | **2.0** | 0.63 | 3.11 | 8.55 | 17.31 |
|  | **3.0** | 0.91 | 4.46 | 11.96 | 23.32 |
|  | **4.0** | 1.47 | 7.05 | 18.09 | 33.09 |
|  | **5.0** | 1.81 | 8.59 | 21.47 | 37.97 |
|  | **6.0** | 1.78 | 8.44 | 21.15 | 37.52 |
|  | **7.0** | 1.95 | 9.20 | 22.77 | 39.77 |

Positive predictive values derived from the sensitivity and specificity of the risk score applied to the cumulative risk of CRC for the age interval using Bayes theorem.
